# Optimization and Application of Bioflocculant Passivated Copper Nanoparticles in the Wastewater Treatment

**DOI:** 10.3390/ijerph16122185

**Published:** 2019-06-20

**Authors:** Nkosinathi Goodman Dlamini, Albertus Kotze Basson, Viswanadha Srirama Rajasekhar Pullabhotla

**Affiliations:** 1 Department of Biochemistry and Microbiology, University of Zululand, Private Bag X1001, KwaDlangezwa 3886, South Africa; BassonA@unizulu.ac.za; 2 Department of Chemistry, University of Zululand, Private Bag X1001, KwaDlangezwa 3886, South Africa

**Keywords:** copper nanoparticles, flocculation activity, removal efficiency, wastewater treatment

## Abstract

Nanotechnology offers a great opportunity for efficient removal of pollutants and pathogenic microorganisms in water. Copper nanoparticles were synthesized using a polysaccharide bioflocculant and its flocculation, removal efficiency, and antimicrobial properties were evaluated. The synthesized nanoparticles were characterized using thermogravimetry, UV-Visible spectroscopy, Fourier-transform infrared spectroscopy (FT-IR), powder X-ray diffraction, scanning electron microscope (SEM), and transmission electron microscope (TEM). The highest flocculation activity (FA) was achieved with the lowest concentration of copper nanoparticles (0.2 mg/mL) with 96% (FA) and the least flocculation activity was 80% at 1 mg/mL. The copper nanoparticles (CuNPs) work well without the addition of the cation as the flocculation activity was 96% and worked best at weak acidic, neutral, and alkaline pH with the optimal FA of 96% at pH 7. Furthermore, the nanoparticles were found to be thermostable with 91% FA at 100 °C. The synthesized copper nanoparticles are also high in removal efficiency of staining dyes, such as safranin (92%), carbol fuchsine (94%), malachite green (97%), and methylene blue (85%). The high removal efficiency of nutrients such as phosphate and total nitrogen in both domestic wastewater and Mzingazi river water was observed. In comparison to ciprofloxacin, CuNPs revealed some remarkable properties as they are able to kill both the Gram-positive and Gram-negative microorganisms.

## 1. Introduction

Surface water has different constituents which need to be removed for the supply of potable water systems. These constituents that need to be removed can be subdivided into colloidal solids, settleable suspended solids, and dissolved solids [1,2]. The treatment of drinking water mostly consists of flocculation, filtration, and disinfection processes. The dosing of a coagulant in water results in the destabilization of negatively charged particles, which results in bigger flocs through a flocculation process, which is known as coagulation [3]. Efficient flocculation is described as the gentle water movement, which results in the gathering of small flocs to form larger masses better suitable for removal by clarification and finally by filtration. Flocculation is an extensively used technology in wastewater treatment, due to its convenience, cheapness, energy-efficiency, easy running, and environmental friendliness [4].

In urban areas, atmospheric deposition, vegetation, and fertilizers are a primary source of nutrients. Nutrients load can also be significantly added through maintenance, construction, and soil management through the addition of fertilizer in the process of reviving injured vegetation [5]. Moreover, nitrogen can result in nitrate and nitrite ions that are part of the nitrogen cycle and occur naturally. Nitrite in excess concentrations can cause diseases. In fish, nitrites can produce a condition known as “brown blood disease” and in warm-blooded animals, it directly reacts with hemoglobin to produce methemoglobin [6].

Industrialization is good for the economic growth of any country. However, industrial growth in many instances produces a huge amount of waste, which ends up reaching the water bodies if untreated. The global growth of waste-producing industries is increasingly becoming a problem to the environment and it is a major contributing factor in water pollution [7]. Synthetic flocculants seem to be the only option. Some of these synthetic flocculants have been found to cause adverse effect to human health and the environment [8]. The process of using flocculants to aggregate and floc colloidal and freely suspended particles is called flocculation (IUPAC, 1997) [9]. Flocculants have various biotechnological applications in wastewater, drinking water treatment, the removal of dye solutions, inorganic solid suspensions, and industrial downstream processing [10]. There are inorganic flocculants, organic flocculants, and naturally occurring flocculants, such as polyaluminum chloride and polyacrylamide derivatives, respectively [2]. Naturally occurring flocculants are usually in a form of extracellular polymers (EPS) of proteins, glycoproteins, lipids, glycolipids, polysaccharides (such as cellulose), and nucleic acids [11].

Nanoparticles are part of an emerging field of science that refer to the synthesis and development of numerous nanomaterials with size ranges of 1–100 nm [12]. Specifically, copper nanoparticles (CuNPs) have attracted a considerable amount of interest from researchers due to the properties they possess such as low production cost and antibacterial effectiveness. In comparison with precious metals, for instance, gold, silver, or palladium, copper nanoparticles have a high surface-area-to-volume ratio, catalytic activity, optical, and magnetic properties [13]. However, their immediate oxidation when exposed to air becomes the main challenge in their preparation and preservation. The synthesis of these material in an inert atmosphere such as nitrogen or argon have been used by several researchers to overcome this problem. Reducing agents and capping agents that are used in the synthesis have been found to have toxic effects and are very expensive. To overcome the toxicity resulting from the use of reducing agents, we synthesized CuNPs using a green and environmentally friendly route, in this synthesis we have employed bioflocculant in the chemical reduction process. A bioflocculant can function both as a reducing and protecting agent for the copper nanoparticles. This makes the process nontoxic, economical, and friendly to the environment [14]. The current paper is aimed at the optimization and application of bioflocculant passivated copper nanoparticles in wastewater treatment. The impact imposed by chemicals in the treatment of water do not only affect animals and humans, but also has a negative impact on environment. The current study contributes towards an environmentally friendly and eco-efficient method with marginal safety to human and animal’s health. The as-synthesized composite nanomaterial has shown its applications in the treatment of antibiotic resistant microorganism, dye degradation, and water treatment.

## 2. Materials and Methods

### 2.1. Synthesis of Copper Nanoparticles

In total, 3 mM copper sulphate solution was prepared in distilled water. After which, 0.5 g of pure bioflocculant was added and the mixture was shaken for 5–10 min in a shaking incubator at room temperature. The mixture was left standing for 24 h at room temperature and the resulting precipitate was collected by centrifugation at 8000 rpm for 15 min at 4 °C. The synthesis of copper nanoparticles was monitored through physical observation and characterization. Control was a copper sulphate solution without bioflocculant [15].

### 2.2. Characterization of As-Synthesized Copper Nanoparticles 

Thermogravimetric analysis measurements were performed on the as-synthesized copper nanoparticles using a Perkin-Elmer Thermal Analysis Pyris 6 TGA (PerkinElmer, Inc., Waltham, Massachusetts 02451, USA). The nitrogen gas flow rate was maintained at 40 cc/min with ramping of the temperature from 22 to 900 °C at 10 °C/min. The optical measurements of the as-synthesized copper nanoparticles were carried out using Varian Cary 50 Conc UV–Vis spectrophotometer (Agilent Technologies, California 95051, USA). The UV-Vis analysis on the dilute samples of the nanoparticles were conducted using the wavelength region 300–700 nm operated using the 1 nm resolution and at room temperature conditions. Fourier transform-infrared (FT-IR) analysis was performed on the bioflocculant and as-synthesized nanoparticles using the Tensor 27, Bruker FT-IR spectrophotometer with a resolution of 4 cm^−1^ in the range of 4000–200 cm^−1^. The pure dry samples were ground to fine powder and were used in the analysis to identify the functional groups present [15]. 

JEOL JSM-6100 microscope equipped with an energy-dispersive X-ray analyzer (EDX) (JEOL USA, Inc., Peabody, Massachusetts 01960, USA) was used for morphological study of the as-synthesized copper nanoparticles. The SEM images were taken using the Tungston (W) filament operated at an emission current of 100 µA and an accelerating voltage of 10 kV. The dry samples of small quantities were placed on copper stubs using double-sided carbon tape and coated with the carbon with the help of a JEOL vacuum evaporator. The working distance was maintained at 5–10 nm. JEOL JSM 6100 SEM with Bruker Quantax Esprit software (JEOL USA, Inc., Peabody, Massachusetts 01960, USA) was used for the EDX measurements. JEOL 1010 transmission electron microscope (JEOL USA, Inc., Peabody, Massachusetts 01960, USA) was used to obtain the TEM images. The specimens were prepared by placing a drop of diluted sample on the copper grid (150 mesh size). The samples were viewed at 100 kV accelerating voltage and digital images were captured using Megaview III camera. Bruker D8 Advance diffractometer equipped with Cu-Kα radiation (λ = 1.5406 Å) was used to study the crystallinity of the as-synthesized copper nanoparticles. The dry powder samples were placed on the sample holder and diffraction patterns were recorded at room temperature using 40 kV and 40 mA [14].

### 2.3. Test for Flocculation Activity of Copper Nanoparticles

A solution of kaolin clay consisting of 4 g in 1 L distilled water was prepared. Then, 100 mL of the prepared solution was transferred into 3 separate 150 mL conical flask; thereafter, 2 mL (0.2 mg/mL) solution of the nanoparticles was added, 3 mL CaCl_2_ (1%) solution was also added, and the mixture was shaken for 1 min and transferred to 100 mL graduated measuring cylinders. The mixture was left to stand for 5 min before supernatant was taken for analysis [16]. Flocculation activity was calculated according the following equation:

(1) $$\mathrm{Flocculation}\;\mathrm{activity},\;\mathrm{FA}\%=\frac{\left[A-B\right]}{A}\times 100$$

*A* is optical density of control at 550 nm and *B* is optical density of sample at 550 nm.

### 2.4. Optimization of Copper Nanoparticles in Flocculation Activity

Different parameters such as dosage, cations, pH, temperature, and speed were evaluated for their effect on flocculation activity. For dosage effect, 0.2–1.0 mg/mL range was used, after obtaining the dosage that resulted in the maximum flocculation activity. Different cations were tested including monovalent, divalent, and trivalent cations. Furthermore, different pH was evaluated in the range of 3–12 and thereafter, the thermo stability of the nanoparticles was evaluated in the temperature range of 50–100 °C. Finally, the effect of speed was conducted by varying speed from 0–220 rpm. These were all done to ascertain which conditions favor the optimal flocculation activity [17]. All data were collected in triplicates with mean and standard deviation values determined where differences were considered significant at 0.05 at confidence level (*p* > 0.05) by the use of Graph Pad Prism™ version 6 and data were analyzed using One-way of variances (ANOVA).

### 2.5. Removal Efficiency of Dyes by Copper Nanoparticles

Decolorization experiments were performed, where 1 mL of copper nanoparticles was added into a 50 mL dye solution (4 g/L), after which the mixture was shaken for a minute and was left to stand for 10 min at room temperature. Test dyes (g/L) included (safranin, methylene blue, carbol fuchsine, malachite green). The supernatant was taken for analysis using UV-VIS Spectrophotometer after the mixture had been stirred for a minute and allowed to settle for 10 min. The absorbance of each sample was measured at the maximum wavelength of each dye. Decolorization efficiency was calculated using the formula below:

RE (%) = [(C_0_ − C)/C_0_] × 100 (2)

where C_0_ is the initial value and C is the value after the flocculation treatment. 

Based on the initial and final dye concentrations, it is important to measure residual concentration of the dye in the samples after treatment [18].

### 2.6. Application of Nanoparticles in the Treatment of Wastewater

Wastewater samples were collected from the Vulindlela water treatment plant, Tendele coal mine, and Mzingazi River to ascertain removal efficiency (RE) of pollutants by nanoparticles in the water sample. NaOH or HCl was used for adjusting pH value when necessary. The wastewater sample was then poured into a 100 mL beaker and 2 mL of 0.2 mg/mL nanoparticles were added, and the mixture was stirred at the designed agitation speed for 10 min, and then allowed to stand for 30 min. The supernatant was taken for analysis using spectrophotometer at 680 nm and the residual Chemical oxygen demand (COD), Biochemical oxygen demand (BOD), total nitrogen, and phosphate were measured. To measure the total nitrogen, sulphate, COD, and BOD, test kits were used following manufacturer’s protocol. The removal efficiency (RE) of the pollutants was calculated using a same equation as indicated above.

### 2.7. Antimicrobial Activity Test for Synthesized Nanoparticles

Test bacteria of choice were first resuscitated by inoculation into the sterile nutrient broth and incubated at 37 °C overnight. After which, 1 mL from each culture was inoculated into separate test tubes containing 9 mL of sterile nutrient, the test tubes were labelled with *Escherichia coli*, *Bacillus pumilus*, *Citrobacter freundii*, and *Klebsiella pneumoniae*. The culture was then incubated overnight at 37 °C. The absorbance of each organism was then determined at 600 nm using a UV-visible spectrophotometer to ascertain the turbidity of each organism. The turbidity of all the organisms was then adjusted using fresh sterile nutrient broth to attain an absorbance of 0.1–0.5, which is within McFarland accepted standard.

#### 2.7.1. Minimum Inhibitory Concentration (MIC)

A method described by Maliehe et al. [19] was adopted. The minimal inhibitory concentration (MIC) of the synthesized copper nanoparticles was determined. MIC is described as the lowest concentration of the CuNPs required to inhibit microorganisms. Quantitative determination of the synthesized nanoparticles was achieved through the use of 96-well plates. All the wells of the 96-well plates were inoculated with 50 μL of sterile nutrient broth. Then, 0.2 g of CuNPs was dissolved into 2 mL of distilled water. The solution of CuNPs (50 μL) was then poured into the first row of 96-well plates containing nutrient broth, and mixed. A 3-fold dilution was then performed whereby (50 μL) from row A was taken to row B of the 96-micro-well plates and it was mixed again, and another (50 μL) was taken from row B to other subsequent rows until all the wells had the CuNPs in different concentrations. The (50 μL) in the last column was discarded so that the total volumes of all the 96 wells is (50 μL). Selected bacteria strains were then added (50 μL) into corresponding wells. The antibiotic Ciprofloxacin (40%) was used as a positive control, while distilled water was used as a negative control.

The plates were then incubated at 37 °C overnight. P-iodonitrotetrazolium violet (INT) solution was used as an indicator after the incubation period. Then, 40 μL of 0.2 mg/mL INT solution was added to each well and further incubated at 37 °C for 30 min. The presence of a reddish color indicated the reduction of INT to formazan by a metabolic active microorganism. The absence of the reddish color (clear) was an indication of inactivity of microorganisms since INT was not broken down to form formazan. All the tests were conducted in triplicates and mean values were taken.

#### 2.7.2. Minimum Bactericidal Concentration (MBC) 

MBC was determined by using the agar dilution method. A loop full of the culture of each strain from the wells that indicated no color change was streaked on a Muller Hilton nutrient agar. The plates were incubated at 37 °C for 12 h. The lowest concentration of CuNPs that exhibited the complete killing of the test organisms was considered as the MBC [20]. 

## 3. Results and Discussion

### 3.1. Characterization Results of Purified Bioflocculant and As-Synthesized Copper Nanoparticles 

Three phases were noticed in the thermogravimetric analysis of as-synthesized copper nanoparticles. The first phase is observed from 40–120 °C due to the loss of moisture, the second phase appeared around 150–200 °C and could be attributed to the decomposition of polymer, and the third phase was observed at higher temperatures due to more weight loss from the samples [15]. The UV-Visible spectra of the bioflocculant and as-synthesized copper nanoparticles showed peak maxima at around 290–300 nm that could be ascribed to Cu nanoparticles formation [15]. Different functional groups viz., hydroxyl (-OH) group and amine (-NH_2_) group were present in the molecule representing the bands at 3276 cm^−1^ (bioflocculant) and 3408 cm^−1^ (copper nanoparticles). The weak band at 2182 cm^−1^ and the peak located at 1670 cm^−1^ in both samples indicates the aliphatic bonds and amide group. The presence vibrational band for Cu-O bonds can be observed at 596 cm^−1^. The presence of saccharide derivatives can be observed as peaks between 1000–1100 cm^−1^ [15].

SEM-EDX results illustrated the presence of O, C, P, Ca, Cl, Na, K, Mg, and S elements in the purified bioflocculant. The presence of O, Cu, P, Mg, Ca, Cl, and S elements in the as-synthesized nanoparticles represent the passivation of the copper nanoparticles with the bioflocculant [15]. The SEM images shows the amorphous nature of the purified bioflocculant and copper nanoparticles. The larger particle sizes can be observed for the bioflocculant passivated copper nanoparticles. The copper nanoparticles appear to be close to spherical in shape and are aggregated [15]. The TEM image revealed the average particle size of 54 nm and close to spherical shape. The particle size was found to be ~53.56 nm using the Scherrer equation: D = (Kƛ)/(β cos θ) [15]. Powder X-ray diffraction patterns for as-synthesized copper nanoparticles showed the characteristic peaks at around 33° and 47° 2θ corresponding to (111) and (220) planes of the fcc structure, which are in good agreement with the copper standard (JCPDS 04-0836) [15].

**Figure 1:**
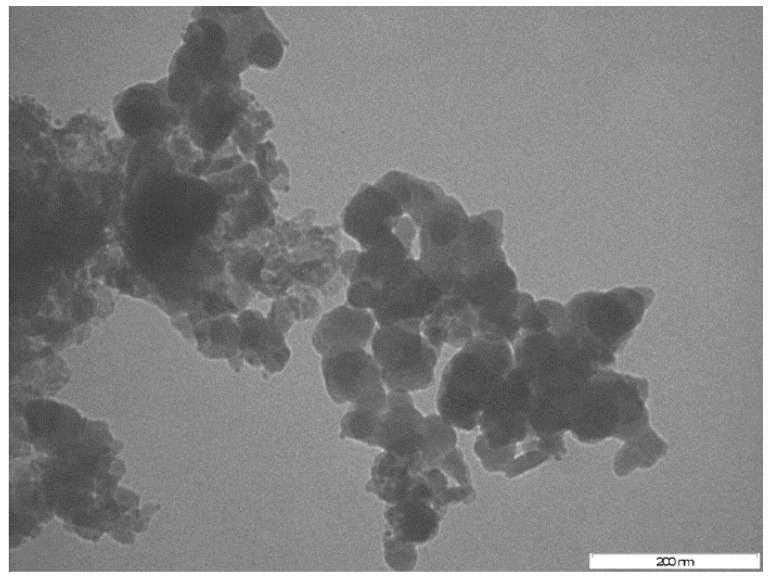
TEM (transmission electron microscope) image of as-synthesized copper nanoparticles at 200 nm scale.

**Figure 2:**
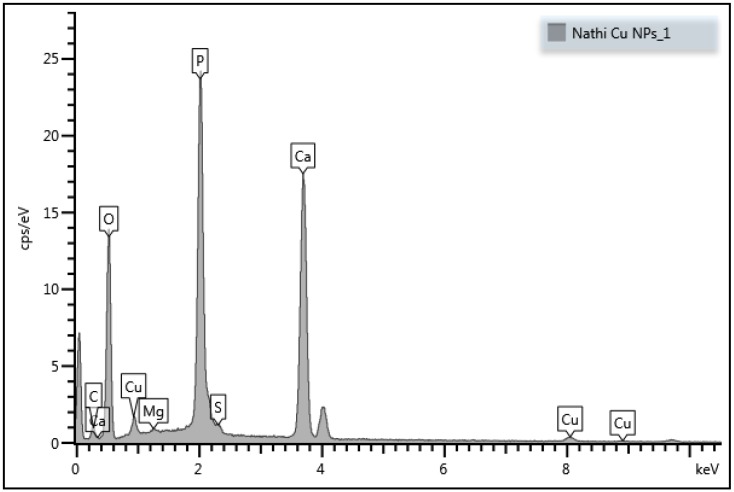
SEM-EDX (scanning electron microscope-energy-dispersive X-ray analyzer) image of as-synthesized copper nanoparticles.

Figure 1 and Figure 2 show the TEM and SEM-EDX images of synthesized copper nanoparticles. From the TEM image, it is evident that as-synthesized copper nanoparticles were agglomerated and has close to spherical shape. The EDX results confirms the presence of copper in a sample, which is an indication that the bioflocculant could be used as a capping agent in nanoparticles synthesis. The detailed characterization results of the as-synthesized nanoparticles can be found elsewhere [15].

### 3.2. Effect of Dosage on Flocculation Activity of Nanoparticles

**Figure 3:**
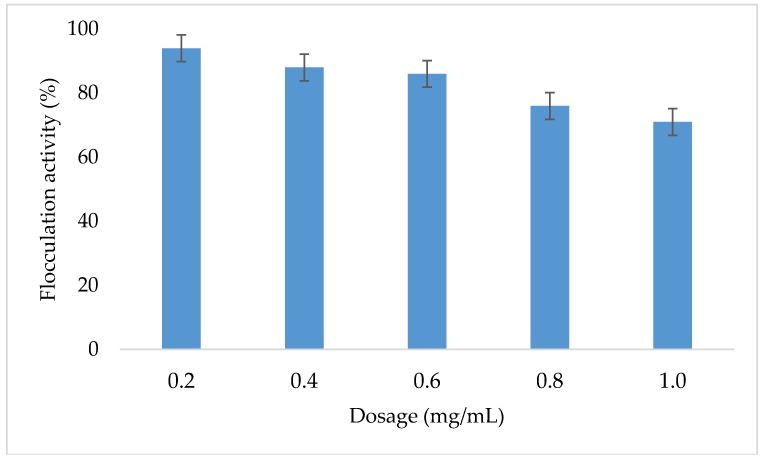
Effect of copper nanoparticles dosage on flocculation activity.

Figure 3 represents the results obtained during the determination of the effect the copper nanoparticles concentration on flocculation activity. The flocculation activity of copper nanoparticles decreased proportionally with the increase in dosage concentration.

The amount of nanoparticles powder required for optimal flocculation is called dosage size. The different concentration of copper nanoparticles (CuNPs) solution was prepared and its flocculation activity was evaluated. Various concentrations were prepared by dissolving different amounts of CuNPs powder in the concentration of 0.2, 0.4, 0.6, 0.8, and 1 mg/mL in distilled water. Each of the dittos was dissolved in 50 mL distilled water. After which 1 L of kaolin clay solution was prepared whereby 4 g of kaolin clay powder was dissolved in a liter of distilled water. Three milliliters of 1% (w/v) CaCl_2_ and 2 mL of CuNPs solution was added into a 500 mL conical flask containing 100 mL of kaolin clay and then agitated for 1 min. The mixture was transferred to a graduated 100 mL measuring cylinder and left to stand for 5 min. The supernatant was taken for analysis in a spectrophotometer with a wavelength of 500 nm. As depicted in Figure 1, the nanoparticles flocculate best at a low dosage as the highest flocculation activity was achieved at 0.2 mg/mL with the flocculation activity of 96%. The increase in dosage concentration resulted in a decrease in flocculation activity. The highest flocculation activity was achieved at the lowest dosage of 0.2 mg/mL. According to Peng et al. [21], excessive addition of the flocculants results in destabilized kaolin particles suspension, which in turn results in repulsion of negatively charged kaolin particles. The increase in dosage concentration results into decrease in flocculation activity. This could be due to the blocking of the binding site of kaolin particles by the excess flocculating agent [20]. Most literature reports on the low dosage with the concentrations between 10–50 mg/L are more effective in flocculation activity [21]. Contrary to these findings, the bridging phenomena could not be effectively formed when the dosage of the bioflocculant was too low [22].

### 3.3. The Effect of pH on Flocculation Activity of Copper Nanoparticles

**Figure 4:**
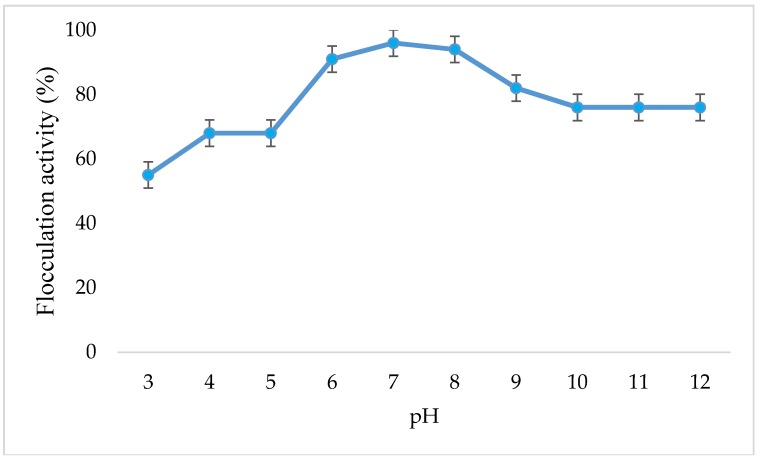
Effect of pH on the flocculation activity of synthesized copper nanoparticles.

Figure 4 shows the effect of pH on flocculation activity of copper nanoparticles. CuNPs work best in weak acidic, neutral, and alkaline pH. Key factors that influence the flocculation process include the pH of the reaction mixture [23]. The flocculant charge status and surface characteristics of the colloidal particles may be altered by pH, which in turn may affect flocculating efficiency [24]. Different flocculants have been reported to produce flocculating efficiency with optimal activity at varying pH values. NaOH and HCl were used to adjust kaolin solution’s pH whenever it was necessary.

Figure 4 shows a strong flocculation activity that can be observed over a wide range of pH 3–12. The maximum flocculation activity of 96% was achieved at neutral pH 7 implying that the adjustment of pH would not be necessary for achieving high flocculation with these nanoparticles. However, at acidic pH, low flocculation activity was observed (55% at pH 3). This might be due to the bioflocculant, which showed different electric states at different pH, thus affecting the bridging efficiency of the bioflocculant for clay powder [25]. At alkaline pH, the flocculation activity remained constant from pH 10–12 with flocculation activity above 75%. A method described by Yu-sen et al. [26] was adopted to ascertain the presence of copper ions in the flocculated water sample; whereby, 50 mL of flocculated sample was centrifuged at 4000 rpm for 30 min. Subsequently, 50 mL of the supernatant containing soluble copper was removed 0.05 M of ammonium was added and there was no presence of blue precipitate observed, which indicated the absence of copper ions.

### 3.4. The Effect of Cations on Flocculation Activity of Copper Nanoparticles

Table 1 represents the results obtained during the determination of the effect of cations on flocculation activity of CuNPs. Synthesized CuNPs are independent cations, as the flocculation activity was above 95% without addition of cations.

The effect of cations on flocculation activity of CuNPs was investigated and all cations enhanced the flocculation activity. The synthesized copper nanoparticles without the addition of cations control was the second highest with a flocculation activity of 96%. According to a study conducted by Li et al. [27] trivalent, divalent, and monovalent cations have stimulated the adsorption of bioflocculant on the suspended kaolin particles by decreasing the negative charge of both the polymer and particles. This stimulation process of flocculation activity was observed in a bioflocculant produced by Bacillus licheniforms and Bacillus circulans when Al^3+^ and Ca^2+^ were used [28]. Cations neutralize and stabilize the negative charge of the functional groups of colloidal kaolin particles in solution and the bioflocculant [27]. Synthesized copper nanoparticles are more effective without the addition of cation with 96% flocculation activity. This makes the synthesized nanoparticles commercially valuable.

### 3.5. The Temperature Effect on Flocculation Activity of Copper Nanoparticles

**Figure 5:**
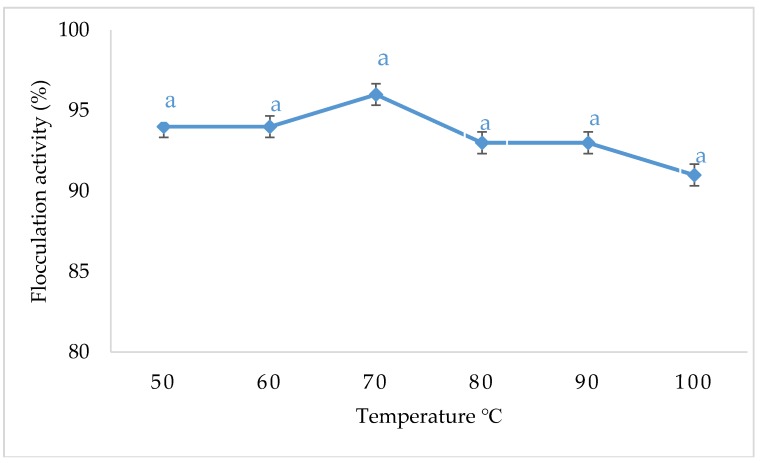
Effect of temperature on flocculation activity of CuNPs. Percentage flocculating activities with letter (a) are significantly (*p* < 0.05) different.

Figure 5 shows the effect of temperature on flocculation activity of copper nanoparticles. CuNPs were found to be heat stable with a flocculation activity above 90%.

The relationship between temperature and flocculating efficiency of the synthesized nanoparticles was examined at a temperature range from 50–100 °C [29]. The synthesized nanoparticles were subjected to different temperatures using a water bath for 30 min; this was done to ascertain its stability when subjected to higher temperatures. The synthesized nanoparticles retained over 90% flocculation activity, suggesting that the nanoparticles were very stable. The highest flocculation activity was achieved below 70 °C, increase in temperature resulted in slightly decreased flocculating efficiency, but the difference was not significant in terms of statistical analysis. Therefore, it was deduced that the nanoparticles were thermo-stable, and its flocculation activity was not affected when the temperature was elevated. The presence of protein or peptide in the structure of a bioflocculant was generally linked to its sensitivity to heat, while sugars containing bioflocculants were more heat resistant. It can be concluded that the bioflocculant from which the nanoparticles were synthesized contains more sugar than protein [30]. The thermal stability of the synthesized nanoparticles may be due to the hydroxyl group found in the bioflocculant that is responsible in the formation of hydrogen bonds in its structure [9].

### 3.6. The Effect of Shaking Speed on Flocculation Activity of Copper Nanoparticles

**Figure 6:**
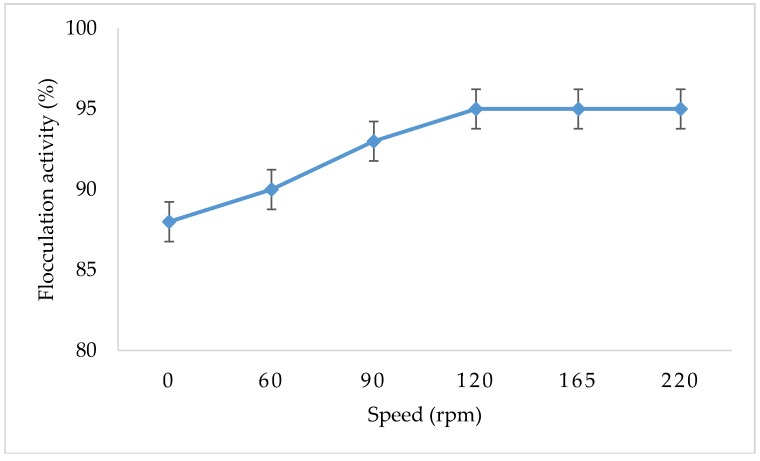
Effect of agitation speed on flocculation activity of CuNPs.

Figure 6 shows the effect of shaking speed on flocculation activity of copper nanoparticles. Speed is one of the parameters which influences flocculation activity. Synthesized CuNPs work well in all ranges of speed evaluated.

The agitation effect on flocculation activity was assed using a shaking incubator. The solution of 100 mL kaolin clay was mixed with 2 mL copper nanoparticles, the mixture was placed inside the shaking incubator for 1 min at different speeds, and flocculation activity was measured. The highest flocculation activity was observed at 220 rpm. However, the nanoparticles worked effectively without agitation with 88% flocculation activity, which indicates that the synthesized nanoparticles can work well without agitation.

### 3.7. The Effect of Copper Nanoparticles on Staining Dye Removal

**Figure 7:**
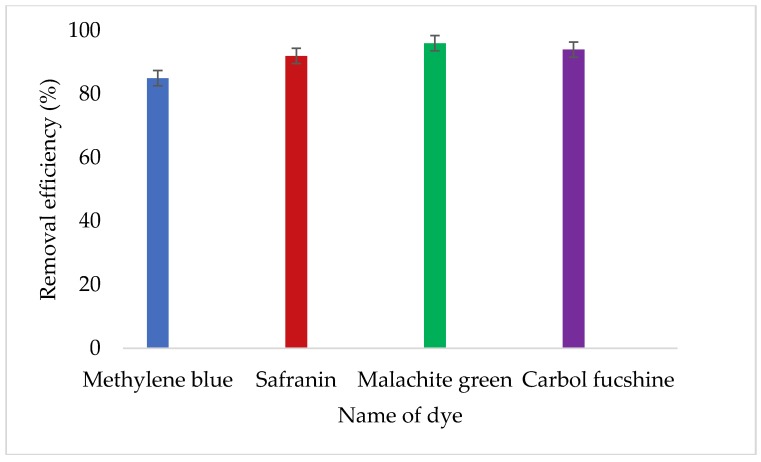
Effect of copper nanoparticles on staining dye removal.

Figure 7 illustrates effect of copper nanoparticles on staining dye removal. The synthesized nanoparticles have a high affinity for all examined dyes with removal efficiency above 85%. These copper nanoparticles are effective to remove dyes in wastewater from different industries like the clothing industry.

The dye removal potential of copper nanoparticles synthesized from a bioflocculant was investigated. Synthesized nanoparticles were able to remove all different dyes. This could be attributed to the aggregation of particles due to bridging and charge neutralization as reported previously [31]. When the particles extend from the surface into a solution for a distance greater than the distance over which the antiparticle repulsion acts, bridging occurs. The synthesized nanoparticles possess huge potential for removing stain in all dyes that were tested. Concentration of nanoparticles remained constant (0.2 mg/mL) and this demonstrated the nanoparticles were effective because removal efficiency was above 80% in all dyes without the addition of cations. This was contrary to the findings reported by Deng et al. [32] where the removal efficiency of dyes directly depended on high concentrations of the bioflocculant. The functional groups present in the polymer must be able to interact with sites on the surface of the colloidal particle in order to be effective [29].

### 3.8. Removal Efficiency of Pollutants in Wastewater

Table 2 shows the removal of nutrients (nitrate, phosphate, and total nitrogen), metals (aluminum and sulphate), and BOD and COD in coal mine wastewater, domestic wastewater, and Mzingazi river water. The nanoparticles showed some remarkable properties for pollutants removal.

In Table 2 above, copper nanoparticles showed the ability to remove P and S in coal mine water. A water sample from a local coal mine was used to ascertain the removal efficiency of synthesized copper nanoparticles. Elements such as P and S were tested. The synthesized copper nanoparticles showed some remarkable ability to remove these elements. The results suggest that copper nanoparticles can be a suitable alternative to replace chemical flocculants. The bioflocculant passivated nanoparticles provide properties such as degradability and friendliness to the environment that the chemical flocculants lack. High levels of COD and BOD in water do not support aquatic life [33]. The presence of N, P, and S in high concentrations in water prompt eutrophication. The application of copper nanoparticles for removal of these pollutants from domestic wastewater, coal mine water, and industrial wastewater was determined. The synthesized nanoparticles had the best removal efficiency for COD and BOD with 93% and 96% removal efficiency, respectively, contrary to the polyamine flocculants, which could remove up to 89% of COD in dye wastewater [2]. The removal efficiency of the copper nanoparticles could be attributed to the surface structure, chemical components, and functional groups. The findings suggest that the synthesized copper nanoparticles possess high potential for industrial application. Furthermore, the effectiveness of CuNPs recommends that they also have the potential to reduce the adverse effects of chemical flocculants being used. The ability of CuNPs to reduce the tested water quality parameters signifies their multi-functionality.

Over the years, there has been a significant increase in nutrients in lakes in response due to the increased discharge of domestic wastewater, pollution from agricultural practices and urban development [33]. Nutrients enrichment, especially nitrate and nitrogen has been considered as the main threat to marine water. This may result in eutrophication, which, in turn, affects the cost of producing potable water. The synthesized copper nanoparticles had effectively removed aluminum, nitrate, and total nitrogen in domestic wastewater. The bioflocculant could not effectively remove these pollutants in wastewater, therefore it can be concluded that copper nanoparticles are more effective. As depicted in Table 2, the synthesized nanoparticles were found to be most effective in BOD removal in domestic wastewater with 96% removal efficiency.

Varying amounts of phosphate can be considered as a result of washing, from farm soil to waterbodies. The presence of phosphate in water stimulates the growth of aquatic plants and plankton, which provide food for fish and this increased growth may increase the population growth of fish and improve overall water quality. Even so, excess of phosphate may cause wild growth of aquatic plants and algal; this may, in turn, result in eutrophication or over-fertilization of receiving waters. As a result, eutrophication or over-fertilization may cause the decay of vegetation and quality of life due to decreased dissolved oxygen levels. Toxicity of phosphate to people and animals could occur from an extremely high levels of phosphate [33]. Table 2 shows the ability of nanoparticles to remove phosphate in the Mzingazi River. The copper nanoparticles had a removal efficiency below 40% when it was tested as per the test kit manufacturer’s protocol. However, when it was left standing for a week, there was a drastic increase in phosphate removal up to 90% as depicted in Table 2. These results suggest that contact time is an important factor in the efficiency of nanoparticles. 

Eutrophication continues to be the primary concern for water quality and phosphorus and nitrogen are the primary nutrients implicated in eutrophication [34]. As can be seen from the results, the copper nanoparticles could remove up to 52% of total nitrogen.

### 3.9. Minimal Inhibitory Concentration, Minimal Bactericidal Concentration in mg/mL for Copper Nanoparticles

Table 3 shows the MIC and MBC of the synthesized copper nanoparticles in comparison with the ciprofloxacin. The synthesized nanoparticles showed some remarkable properties against both Gram-positive and Gram-negative organisms; the least MIC and MBC was observed against *B. pumilus* and *E. coli.*

Copper nanoparticles were evaluated for their antimicrobial effect in comparison with ciprofloxacin, which was used as a control for the experiment. Further, 20 µL ciprofloxacin was used and all the Gram-negative organisms show the inhibitory effect of ciprofloxacin. However, *B. pumilus,* which is Gram-positive, could still grow in the presence of both low and high concentration of ciprofloxacin. On the other hand, copper nanoparticles showed some remarkable properties for inhibiting and killing both the Gram-positive and Gram-negative organisms. Also, 96-well microplate technique was used, against four strains of both Gram-positive and Gram-negative pathogenic organisms. The antimicrobial effect of copper nanoparticles was found to be more prominent on Gram-negative organisms and remarkable properties against Gram-positive were observed, where *B. pimillus* had low MIC of 3.13 mg/mL concentration. Gram-positive bacteria lack the outer membrane and the constituents of the CuNPs are directly in contact with the phospholipid bilayer of the cell [18].

## 4. Conclusions

The synthesized copper nanoparticles showed an excellent flocculation property at a low concentration of 0.2 mg/mL. They flocculate independent of cation, are thermostable, and work at weak acidic, neutral, and alkaline pH. Agitation (shaking speed) is not required for the effectiveness of the synthesized nanoparticles in flocculation. They possess great properties for pollutants removal in coal mine water, domestic wastewater, and river water. Over 89% of COD and BOD removal efficiency was observed for both the coal mine and river water. Furthermore, they are able to remove staining dyes at a low concentration of 0.2 mg/mL and 10 min contact time. The remarkable properties for the removal of staining dyes suggest that the synthesized nanoparticles can be used in removing dye effluents from wastewater. When evaluated for antimicrobial activity against both Gram-negative and Gram-positive bacteria, they were able to inhibit and kill all the tested strains at the lowest concentration of 3.13 mg/mL.

## Figures and Tables

**Table 1:** Effect of cations on flocculation activity of copper nanoparticles (CuNPs).

Cations	Flocculation Activity (%) ± SD
Control Fe^3+^ Mn^2+^ Ba^2+^ K^+^ Li^+^ Na^+^	96 ± 2.08 86 ± 3.74 97 ± 1.52 95 ± 3.46 96 ± 2.88 97 ± 1.15 86 ± 1.15

Values represent mean ± deviation of replicate readings.

**Table 2:** Removal of pollutants in different water samples by copper nanoparticles.

Flocculant	Types of Wastewater	Types of Pollutants in Water	Water Quality before Treatment (mg/L)	Water Quality after Treatment (mg/L)	Removal Efficiency (%)
CuNPs	Coal mine water	Phosphate Sulphate Chemical oxygen demand (COD) Biochemical oxygen demand (BOD)	2.00 0.55 154 123.2	0.3 0.13 11.2 5.0	85 76 93 96
	Domestic wastewater	Phosphate Total nitrogen Nitrate Aluminum Sulphate Chemical oxygen demand (COD) Biochemical oxygen demand (BOD)	7.6 155 20.6 0.86 1.7 2.313 123.2	1.5 17.0 7.7 0.33 0.61 0.654 4.123	80 89 63 62 64 72 96
	Mzingazi River water	Phosphate Total nitrogen Chemical oxygen demand (COD) Biochemical oxygen demand (BOD)	85.7 0.223 3.300 133	7.521 0.108 0.278 15.0	92 52 92 89

**Table 3:** Minimal inhibitory concentration, minimal bactericidal concentration in mg/mL for copper nanoparticles.

Strains of Bacteria	Ciprofloxacin		CuNPs	
	Minimal Inhibitory Concentration (MIC)	Minimal Bactericidal Concentration (MBC)	Minimal Inhibitory Concentration (MIC)	Minimal Bactericidal Concentration (MBC)
*B. pumilus*	-	-	3.13	6.25
*E. coli*	3.125	6.25	6.25	12.5
*C. freundii*	1.56	3.13	12.5	12.5
*K. pneumoniae*	1.56	3.13	12.5	25.0
